# Single-cell RNA-seq identifies unique transcriptional landscapes of human nucleus pulposus and annulus fibrosus cells

**DOI:** 10.1038/s41598-020-72261-7

**Published:** 2020-09-17

**Authors:** Lorenzo M. Fernandes, Nazir M. Khan, Camila M. Trochez, Meixue Duan, Martha E. Diaz-Hernandez, Steven M. Presciutti, Greg Gibson, Hicham Drissi

**Affiliations:** 1 Department of Orthopaedics, Emory University School of Medicine, Atlanta, GA 30033 USA; 2 Atlanta VA Medical Center, Decatur, GA USA; 3 Center for Integrative Genomics, Georgia Institute of Technology, Atlanta, GA USA

**Keywords:** Gene expression profiling, RNA sequencing

## Abstract

Intervertebral disc (IVD) disease (IDD) is a complex, multifactorial disease. While various aspects of IDD progression have been reported, the underlying molecular pathways and transcriptional networks that govern the maintenance of healthy nucleus pulposus (NP) and annulus fibrosus (AF) have not been fully elucidated. We defined the transcriptome map of healthy human IVD by performing single-cell RNA-sequencing (scRNA-seq) in primary AF and NP cells isolated from non-degenerated lumbar disc. Our systematic and comprehensive analyses revealed distinct genetic architecture of human NP and AF compartments and identified 2,196 differentially expressed genes. Gene enrichment analysis showed that *SFRP1, BIRC5, CYTL1, ESM1* and *CCNB2* genes were highly expressed in the AF cells; whereas, *COL2A1, DSC3, COL9A3, COL11A1,* and *ANGPTL7* were mostly expressed in the NP cells. Further, functional annotation clustering analysis revealed the enrichment of receptor signaling pathways genes in AF cells, while NP cells showed high expression of genes related to the protein synthesis machinery. Subsequent interaction network analysis revealed a structured network of extracellular matrix genes in NP compartments. Our regulatory network analysis identified *FOXM1* and *KDM4E* as signature transcription factor of AF and NP respectively, which might be involved in the regulation of core genes of AF and NP transcriptome.

## Introduction

Intervertebral disc (IVD) degeneration (IDD) is a pathophysiological process and is a common contributor to the development of chronic lower back pain^1–4^. IDD has a significant economic burden, amounting to over one hundred billion dollars annually in direct and associated costs^2^. There are three distinct anatomical regions in IVD- an outer annulus fibrosus (AF) composed of radially arranged collagen filaments that enclose an inner gelatinous nucleus pulposus (NP), as well as a cartilaginous endplate that demarcates the boundary between the IVD and the bony vertebral body^3,5,6^. It is postulated that catabolic and anabolic dysregulation of the cells within the AF and NP compartments play a pivotal role in the pathobiology of IDD^1,7^. These metabolic changes can alter the composition of the extracellular matrix (ECM) in the NP and AF compartments^1,8^ that may, in turn, affect the load-bearing and mechanical properties of the IVD leading to degeneration.

The IVD is predominantly avascular, with vasculature limited to the cartilaginous endplate and outer annulus fibrosus^6,9,10^. The environment within the NP is largely hypoxic, and the cells of the NP rely on diffusion of oxygen and nutrients through the endplate^9,11^. These differences in the microenvironments of the AF and NP compartments may influence their gene signatures. While initial pioneering work has revealed transcriptomic profiles of degenerating IVDs utilizing bulk RNA sequencing or microarray-based approaches^12–18^, the transcriptomic signatures of non-degenerated healthy human NP and AF cells remain unexplored. Recent studies have sought to identify these compartment-specific gene signatures, but have relied on IVDs obtained from animals^19–26^. While informative, IVDs from rodents, rabbits, and other animals are known to have important physiological differences when compared to human IVDs^27^, and as such, are not always translatable to the human condition. Therefore, understanding the transcriptomic signatures of healthy, non-degenerated human AF and NP compartments can be useful to understand the basic biology of IVDs, help inform the development of novel therapies, and improve the efficacy of both stem cell and tissue engineering-based regenerative therapies for IDD.

In this study, we employed an unbiased single cell (sc)-RNA-seq approach using NP and AF cells isolated from non-degenerated human IVDs with the goal to identify unique gene expression profiles that distinguishes each compartment. The t-Distributed Stochastic Neighbor Embedding (t-SNE) analysis revealed distinct cell clustering of AF and NP that indicates a clear distinction in the transcriptional signatures of AF and NP cells. Functional annotation clustering analysis showed upregulation of specific gene ontology pathways in AF and NP compartments, which may be indicative of compartment-specific functions. Further, regulatory network analyses identified unique transcription factor specific to AF and NP compartment which is believed to play important role in regulating the signature genes of human IVD transcriptome. Our study provide a valuable genetic resource for further exploration of NP and AF compartments of human IVD. To our knowledge, this is the first demonstration of genetic landscape of healthy intervertebral disc components identifying unique molecular signatures, functional clustering and interaction network.

## Results

### NP and AF express cell type-specific markers and segregate into distinct populations

**Figure 1:**
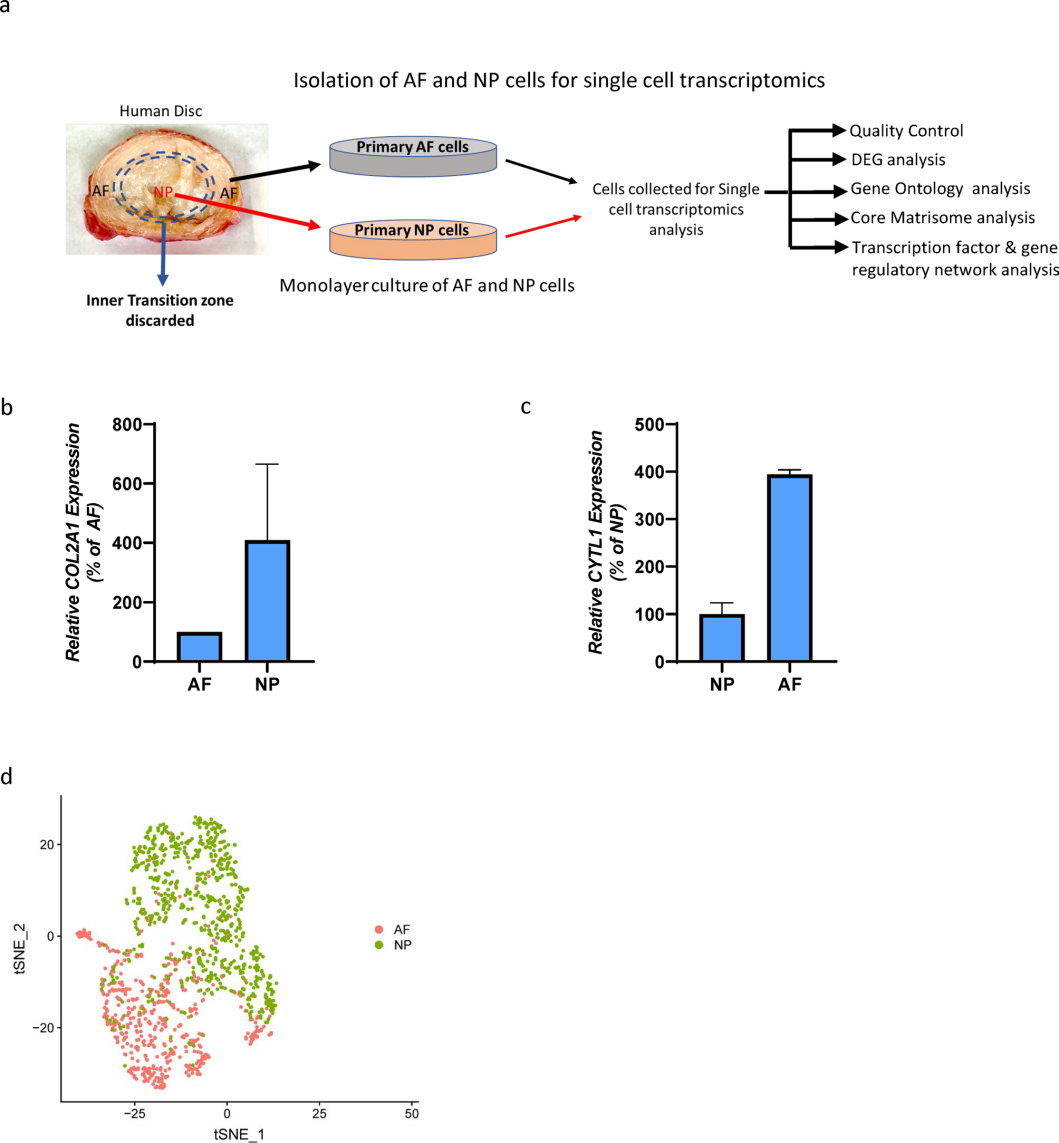
Enrichment of tissue-specific markers and distinct segregation of genes in NP and AF cells. (**a**) Schematic representation of SCT workflow from tissue acquisition to analysis. The IVD image was digitally captured using KodakEasyShare v550 camera and inner dashed circle were traced using MS-Power Point drawing tool to demarcate NP and AF. (**b**) Relative expression of *COL2A1* shows enrichment of the NP marker gene in NP Cells compared to AF cells. (**c**) Relative Expression of *CYTL1* shows higher expression of the AF marker gene in AF cells compared to NP cells. (**d**) tSNE plot showing clear segregation of primary NP and AF cells. The tSNE plot was genereated using Seurat package in R version 3.0.

In order to determine the transcriptomic landscape of NP and AF compartments, we employed a droplet-based scRNA-sequencing approach to evaluate global genome expression profiles of primary NP and AF cells isolated from healthy, non-degenerated human IVDs. A workflow summary is represented in Fig. 1a. The phenotype of the cells in monolayer culture was confirmed by mRNA expression of known marker genes specific to NP and AF (Fig. 1b,c and Supplementary Fig. S1 online). These conditions were then maintained throughout the duration of the study.

Approximately 725 AF and 1,010 NP single cells were analyzed for the expression of 12,323 human genes. We then conducted unsupervised analysis for cell clustering based on transcriptomic profiles using the T-distributed Stochastic Neighbor Embedding (t-SNE) method to define the gene expression heterogeneity of NP and AF cells at the single-cell level. The t-SNE analysis showed that NP and AF cells segregated into two distinct clusters, which is indicative of two transcriptionally discrete populations of cells (Fig. 1d). Taken together, our scRNA-seq data reveal clear transcriptional differences between the two compartments of human IVDs, which is to be expected due to the different developmental origins of these two cell types^28,29^.

### DEG analysis reveals differential gene expression between NP and AF cells

**Figure 2:**
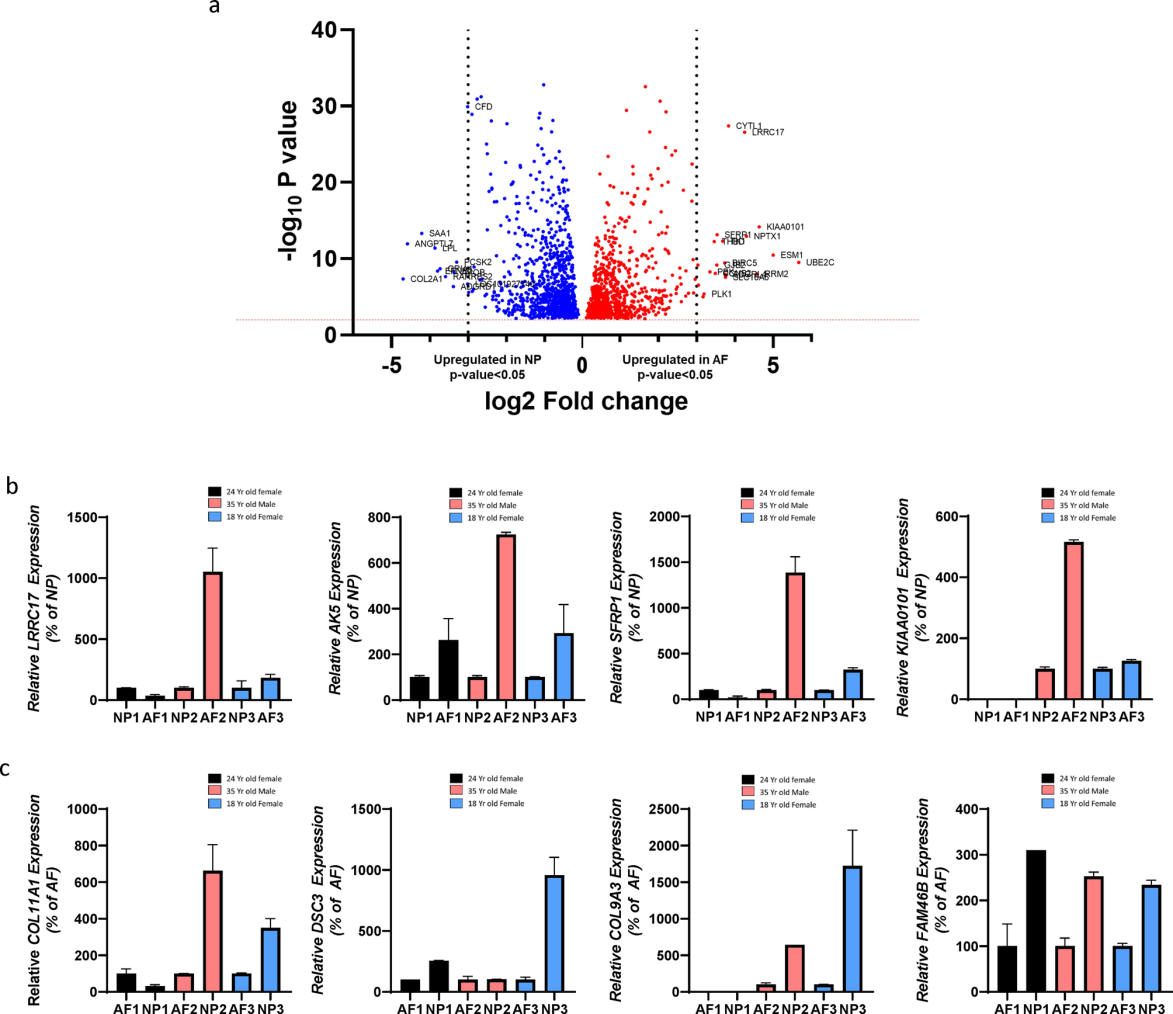
Differential Expression of genes in AF and NP cells. (**a**) Volcano plot depicting differentially expressed genes in AF and NP cells. Red dots represent genes expressed at higher levels in AF cells while blue dots represent genes with higher expression levelsin NP cells. Y-axis denotes − log10 P values while X-axis shows log2 fold change values. Volcano plot was generated using GraphPad Prism version 8.2.0. (**b**) Relative gene expression of LRRC17, AK5, SFRP1 and KIAA0101 in AF cells from 3 human samples expressed as a percentage of expression in NP. (**c**) Relative gene expression of COL11A1, DSC3, COL9A3 and FAM46B genes in NP cells from 3 human samples expressed as a percentage of expression in AF cells. Black, Pink and Blue bars represent gene expression of cells from 24-year-old female, 35-year-old male and 18-year-old female respectively. Bar diagram for gene expression was generated using GraphPad Prism version 8.2.0.

We next sought to determine the expression pattern of the genes that were differentially expressed between NP and AF cells. Our analysis identified 2,196 genes that were differentially expressed between these two cell types, as illustrated in the volcano plot (Fig. 2a). We then identified the most abundantly expressed genes in AF and NP cells based on fold change. *UBE2C, ESM1, KIAA0101,SFRP1, SFRP4, DKK1, WNT5A, APCDD1L, ESM1* and *BIRC5* were amongst the genes that were expressed at higher levels (FDR corrected p-value < 0.05) in the AF compared to NP cells (FDR corrected p-value < 0.05) (Table. 1 and Supplementary Table. S1 online). Conversely, in the NP cells, *COL2A1, KRT8, CD24, DSC3, ANGPTL7, ISM1, NPR3, BIRC3, SAA1, ACAN, FMOD, COMP, ANGPTL7, C2ORF82* and *HIF1A* were some of the genes that were significantly expressed (FDR corrected p-value < 0.05) at higher levels compared to AF cells. (Table. 2 and Supplementary Table. S2 online). The significantly higher expression of these genes suggests that they may contribute to the maintainance and developement of the non-degenerated phenotype of each respective compartment.

**Table 1:** Genes upregulated in AF.

SN	Gene	Mean FDR p-value	Fold change AF
1	*UBE2C*	8.73E−09	50.89
2	*ESM1*	1.09E−09	32.13
3	*KIAA0101*	4.16E−13	24.83
4	*RRM2*	2.34E−07	24.02
5	*NPTX1*	6.30E−12	19.75
6	*LRRC17*	1.09E−24	19.08
7	*CYTL1*	2.16E−25	14.30
8	*SLC16A6*	5.63E−07	13.51
9	*ADGRL4*	2.47E−07	13.45
10	*BIRC5*	1.02E−08	13.32
11	*TK1*	2.19E−11	12.85
12	*SFRP1*	3.94E−12	11.62
13	*GJB2*	1.97E−08	11.56
14	*CCNB2*	1.93E−07	11.24
15	*THBD*	2.55E−11	11.01
16	*PBK*	1.28E−07	10.19
17	*PLK1*	5.71E−05	9.16
18	*MT1G*	1.24E−04	9.00
19	*GAPLINC*	5.03E−06	8.27
20	*PI15*	1.86E−08	8.21

**Table 2:** Genes upregulated in NP.

SN	Gene	Mean FDR p-value	Fold change NP
1	*COL2A1*	9.14E−07	25.98
2	*ANGPTL7*	4.74E−11	23.98
3	*SAA1*	2.80E−12	18.48
4	*LPL*	1.76E−10	14.56
5	*EFNB2*	1.03E−07	13.88
6	*GRIA1*	5.37E−08	13.29
7	*RARRES2*	5.01E−07	12.05
8	*ADGRD1*	7.70E−06	10.42
9	*APOB*	1.58E−07	10.27
10	*PCSK2*	8.99E−09	9.84
11	*CFD*	1.04E−27	8.07
12	*LOC101927740*	3.61E−06	8.07
13	*HPD*	3.85E−05	7.91
14	*SLPI*	9.44E−27	7.46
15	*RASL10B*	2.69E−05	7.41
16	*MAL2*	1.60E−05	7.24
17	*C2orf82*	3.19E−08	7.16
18	*MFAP4*	2.07E−28	6.80
19	*COL9A3*	1.14E−06	6.46
20	*CLEC3B*	1.18E−28	6.30

We further confirmed the expression of a subset of the identified AF- and NP-enriched genes by qPCR using the same 24-year-old female and 35-year-old male samples used for single cell transcriptomic (SCT) analysis, as well as an independent sample obtained from an 18-year-old female that was not analyzed by SCT (all Thompson grade 1 or 2 discs) (Fig. 2b,c and Supplementary Fig.S1,S2a and S2b online). Our analysis confirmed that *LLRC17, AK5, SFRP1* and *KIAA0101* genes were expressed at higher levels in AF cells in at least two of the three samples (Fig. 2b); while *COL11A1, DSC3, COL9A3,* and *FAM46B* were confirmed to be expressed at higher levels in NP cells in at least two of the three samples (Fig. 2c). *KIAA0101* was not detected by qPCR in the 24-year-old sample in both AF and NP cells (Fig. 2b), while COL9A3 was not detected in the 24-year old sample in the AF group (Fig. 2c). Supplementary Fig.S1, S2a and S2b online shows additional genes verified by qPCR in AF and NP cells respectively. These data largely confirm the compartment specific gene signatures identified by our SCT analyses and indicate that the non-degenerated NP and AF compartments contain large numbers of genes that are differentially expressed between the two compartments.

### Single cell trancriptomics (SCT) analysis identifies novel genes in AF and NP cells

**Figure 3:**
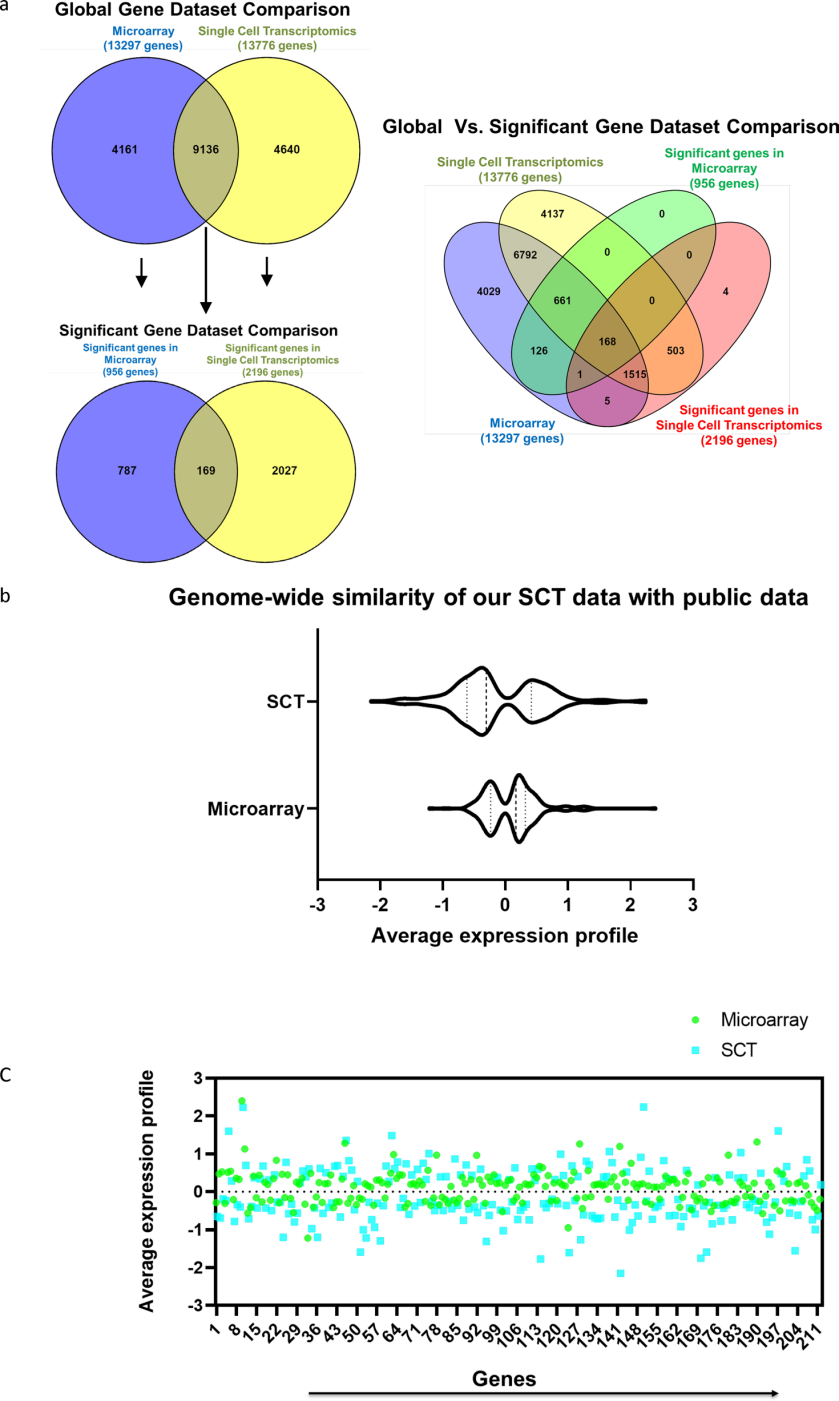
Single cell trancriptomics (SCT) analysis identifies novel genes in AF and NP cells: (**a**) Venn diagram depicting common and unique genes detected by microarray and SCT analysis. (**b**) Violin plot showing genome-wide similarity between microarray and SCT analysis data set. (**c**) Scatter plot displaying the similarity in the expression profile of the 214 common genes between SCT and Microarray data. Each dot represents expression value of a gene in the data set. All the plots were generated using GraphPad Prism version 8.2.0.

In order to determine the sensitivity and robustness of our SCT analysis of AF and NP cells, we compared our data set to publicly available NCBI GEO datasets containing 18 non-degenerated NP and 16 non-degenerated AF human samples (GSE34095, GSE114169, GSE70362, GSE56081, GSE23130). DEG analysis of our SCT dataset detected around 13,776 genes compared to the microarray-based technique, which detected 13,298 genes (Fig. 3a). Among these detected genes, 9,136 were common to both SCT and microarray datasets while 4,161 were unique to the microarray dataset and 4,640 were unique to our SCT dataset (Fig. 3a). Among the total genes detected in both datasets, only 956 were significantly (FDR corrected p-value < 0.05) expressed in the microarray-based GEO dataset compared to 2,196 genes that were significantly (FDR corrected p-value < 0.05) expressed in our SCT analysis (Fig. 3a). Approximately 169 of the significantly expressed genes were common to both datasets. Therefore, our unbiased SCT analysis identified 2,027 novel genes that were not previsouly detected by the microarray-based approach. Our SCT-based dataset showed strong overlap with the publicly-available dataset with a Spearman correlation coefficient of r_s_ = 0.15857, (p = 0.0203) (Fig. 3b,c). These data indicate that our SCT analysis detected more differentially expressed genes between NP and AF cells than microarray, suggesting that SCT analysis may be a more sensitive and robust technique to detect differential gene expression in human IVD cells.

### Gene ontology (GO) analysis reveals pathways enriched in the AF and NP compartments

**Figure 4:**
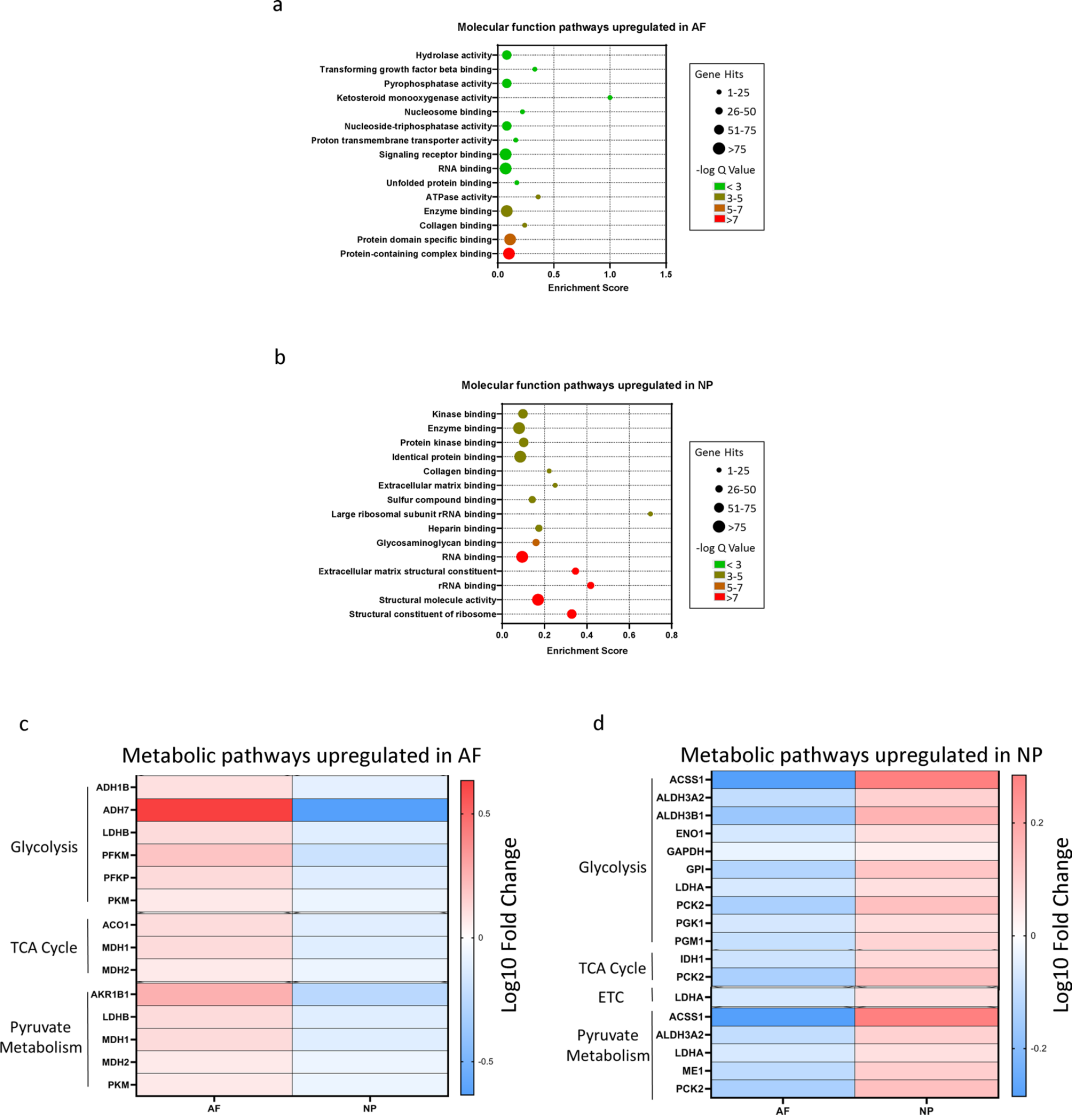
Gene Ontology analysis reveal compartment-specific enrichment of GO pathways in AF and NP cells. (**a**) Advanced bubble plot depicting molecular function-related pathways enriched in AF cells. (**b**) Advanced bubble plot showing molecular function-related pathways enriched in NP cells. Y-axis Labels represent pathway names while and enrichment score is shown on X-axis. The size and color of the bubble represent the number of genes enriched in each pathway and their enrichment significance. The bubble plots were generated using GraphPad Prism version 8.2.0. (**c**) Metabolic pathway analysis of glycolysis, TCA cycle, electron transport chain and pyruvate metabolism pathways in AF. (**d**) Metabolic Pathway analysis of glycolysis, TCA cycle, electron transport chain and pyruvate metabolism pathways in NP cells. Heatmap shows log10 transformed fold change in gene expression and were generated using GraphPad Prism version 8.2.0.

In order to determine the physiological roles of the identified differentially expressed genes, we performed Gene Ontology (GO) enrichment analysis using the ToppGene Suite. Our analysis indicates that the molecular function related GO pathways enriched in AF cells were related to protein-containing complex binding, protein domain specific binding, and ATPase activity (FDR corrected p-value < 0.05) (Fig. 4a). Conversely, the molecular function GO pathways upregulated in NP cells include structural constituents of ribosomes, structural molecule activity and rRNA binding, ECM constituents, and glycosaminoglycan binding and heparin binding (FDR corrected p-value < 0.05) (Fig. 4b). The molecular function pathway categories that were significantly enriched and common to both compartments were collagen binding, enzyme binding and RNA binding (Fig. 4a,b). Considering that the upregulated pathways in the AF and NP are involved in biological processes that are energy-intensive, we next determined the expression level of genes related to the energy generating metabolic pathways that not only supply the bulk of a cell’s energy but also provide reaction intermediates for various anabolic and catabolic processes (Fig. 4c,d). We observed that a greater number of metabolic genes related to glycolysis were expressed at higher levels in the NP cells compared to the AF cells (Fig. 4c,d). *ACSS1, LDHA, GAPDH* and *ME1* were among the genes expressed at higher levels in NP cells compared to AF cells (Fig. 4c,d). *ACSS1* and *LDHA* are common to both glycolysis and pyruvate metabolism. *ME1* plays a crucial role in pyruvate metabolism through the conversion of malate to pyruvate in the cytoplasm, while also generating NADPH in the process^30–32^. The pyruvate produced can then either enter the TCA cycle or be converted to lactate in the cells^33^. Collectively, our data reveal the molecular function pathway-related genes and the major metabolic pathways genes that are differentially expressed in NP and AF cells.

### AF and NP cells show differential expression of ECM-related genes

**Figure 5:**
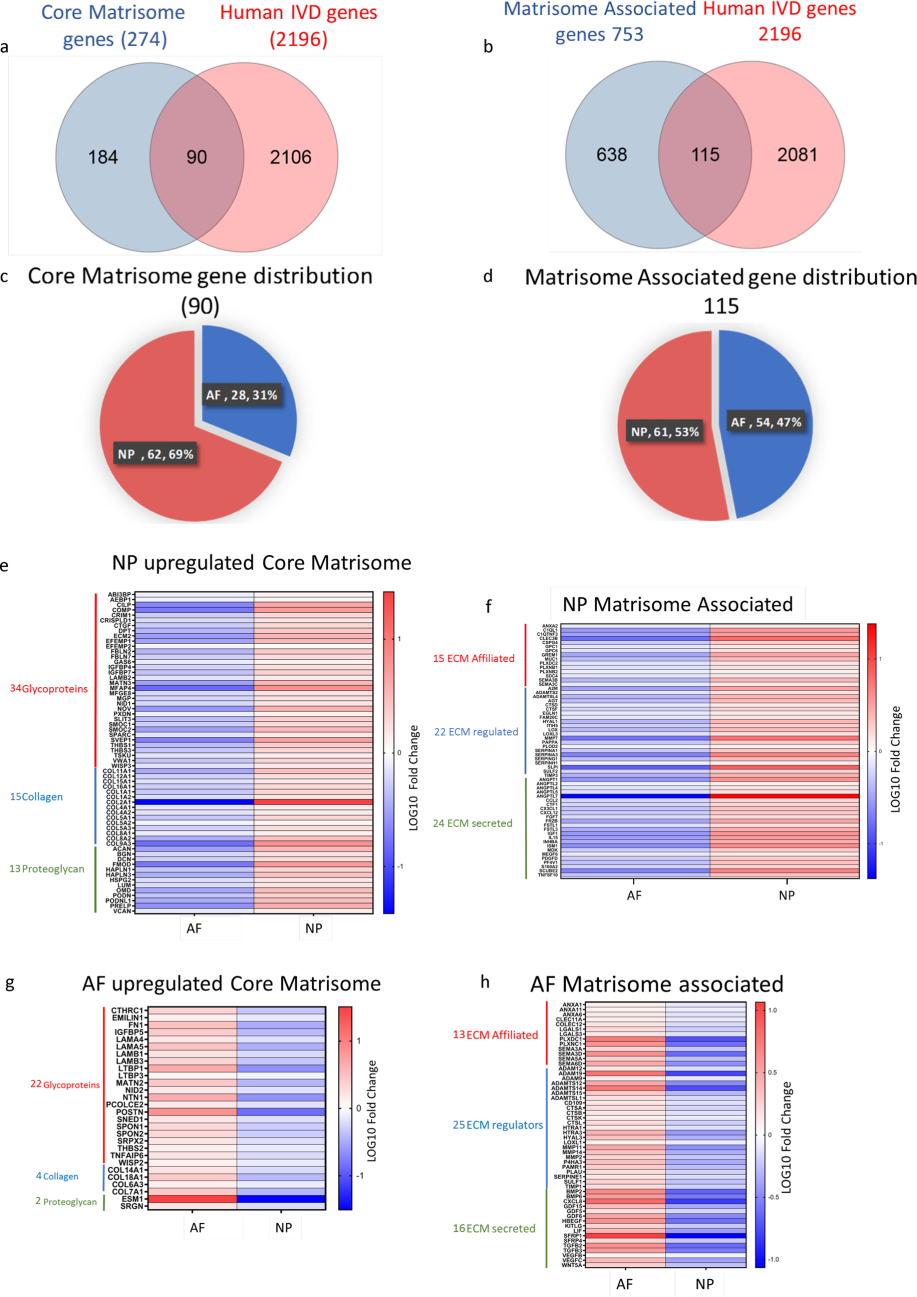
Core matrisome analysis of AF and NP cells show differential expression of genes. (**a**) Venn diagram depicting the total Core matrisome genes, the Core matrisome genes that are present in the IVD, and the total number of differentially expressed genes in our analysis. (**b**) Venn diagram representing the total Core matrisome associated genes, the Core matrisome associated genes that we detect in the IVD and the total number of differentially expressed genes in our analysis. (**c**) Pie chart showing the distribution of Core matrisome genes in AF and NP (AF is shown in red and NP in blue). (**d**) Pie chart showing the distribution of Core matrisome associated genes in AF and NP (AF is shown in red and NP in blue). (**e**) Heatmap showing the differential gene expression profile of Core matrisome genes in human NP cells. (**f**) Heatmap showing the differential gene expression profile of Core matrisome associated genes in human NP cells. (**g**) Heatmap representing the differential gene expression profile of Core matrisome genes in human AF cells. (**h**) Heatmap representing the differential gene expression profile of Core matrisome associated genes in human AF cells. All heatmaps were generated using GraphPad Prism version 8.2.0.

Since many ECM-related molecular fuction patways showed up in our GO enrichment analysis, we next performed a matrisome analysis to determine the core matrisome and matrisome-associated genes differentially expressed in the AF and NP cells. Out of the 2,196 genes differentially expressed between AF and NP cells, 90 were core matrisome genes and 115 were matrisome-associated genes (Fig. 5a,b). Among the core matrisome related genes, 28 were expressed in the AF and 62 in the NP (Fig. 5c). On the other hand, among the matrisome-associated genes, 54 were expressed by AF cells and 61 by NP cells (Fig. 5d). The core matrisome landscape of the NP consisted of 34 ECM glycoprotein genes, 15 collagen genes, and 13 proteoglycan genes (Fig. 5e); conversely, the matrisome-associated genes included 15 ECM affiliated proteins, 22 ECM regulators, and 24 secreted factors (Fig. 5f). The core matrisome of the AF consisted of only 22 ECM glycoprotein genes, 4 collagen genes, and 2 proteoglycan genes (Fig. 5g). The matrisome-associated genes of the AF included 13 ECM affiliated proteins, 25 ECM regulators, and 16 secreted factors (Fig. 5h). Our data suggest that NP cells have higher enrichment of core matrisome and matrisome-associated genes compared to the AF cells.

### *FOXM1* and *KDM4E* transcription factors are among the key regulators of AF and NP gene networks, respectively

We performed an interaction network analysis to identify the functional relationships among the genes specific to the NP and AF compartments. To this end, we evaluated the potential interactions by mapping the NP- and AF-specific genes using the STRING database. We restricted our PPI analysis to only experimentally validated interactions with a combined score of > 0.7, corresponding to highly significant interactions. Our analysis revealed that these genes formed a significant functional network that is involved in several essential biological processes. Network analysis of the protein–protein interaction (PPI) network for NP revealed 0.206 clustering Coefficient, 3 Connected Component, Network Diameter of 9, Network Radius of 2, 0.177 Network Centralization, 3,408 (66%) Shortest Paths, 3.806 Average Neighbors, Characteristic Path Length of 3.30, 0.054 Network Density and 0.906 Network Heterogeneity (Supplementary Table. S3 online). Network analysis of AF cells also demonstrated similar topological properties, however the interaction network for AF was denser than that of NP as revealed by higher number of neighbors (8.918) and higher network density (0.092) (Supplementary Table. S4 online).

**Figure 6:**
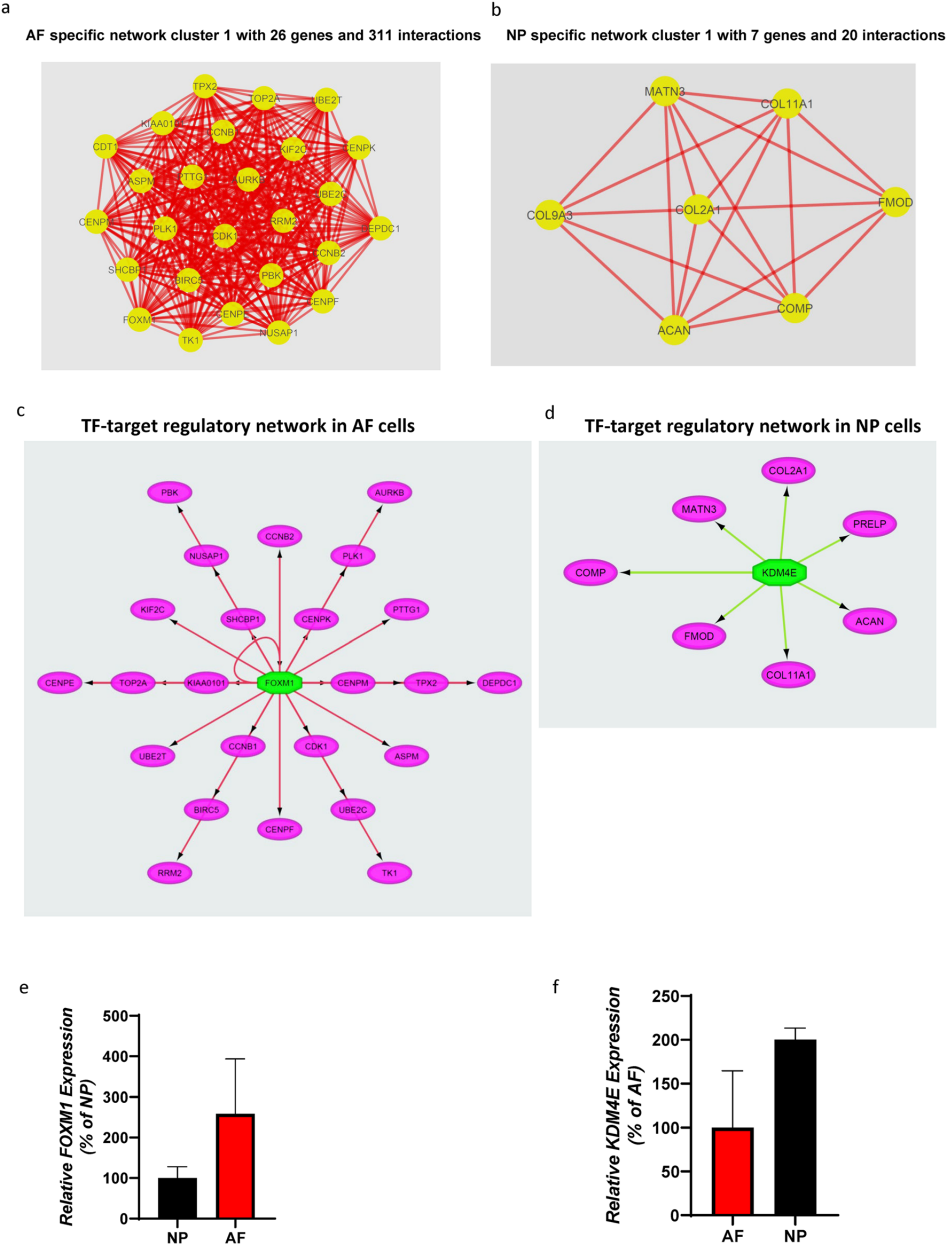
Regulatory network analysis identifies AF and NP-specific TF-targeted regulatory networks. (**a**) AF specific network cluster with 26 genes and 311 interactions. Red lines represent interactions, and yellow circles represent genes. (**b**) NP-specific network cluster with 7 Genes and 20 interactions. Red lines represent interactions, and yellow circles represent genes. The network clusters were made using Cytoscape (version: 3.7.1). (**c**) TF-targeted regulatory network showing FOXM1 and the genes regulated by it in the AF. (**d**) TF-targeted regulatory network showing KDM4E and the genes regulated by it in the NP. The regulatory networks were generated by iRegulon (version 1.3) package using Cytoscape (version: 3.7.1). (**e**) Relative expression of *FOXM1* expressed as a percentage of expression in NP cells. (**f**) Relative expression of *KDM4E* expressed as a percentage of expression in AF cells. Bar diagram for gene expression was generated using GraphPad Prism version 8.2.0.

To identify clusters in the network, we performed a subnetwork analysis using MCODE. Our cluster analysis of AF cells resulted in 4 clusters, which included 38 nodes and 325 edges (Supplementary Table. S5 online). In the NP cells, there were only 2 clusters, which included 14 nodes and 29 edges (Supplementary Table. S6 online). We selected cluster 1 from AF and NP cells for further analysis as it was the most significant and dense cluster in both compartments. Network cluster 1 in the AF possessed a score of 24.9 along with 26 genes that had 311 interactions, indicating the high density of the interaction network. Genes in AF cluster 1 primarily included *FOXM1*, *DEPDC1*, *TK1*, *SHCBP1*, *CDK1*, *UBE2C*, *CCNB2*, *CCNB1*, *CENPF*, *CENPE*, *PLK1*, *BIRC5*, *PTTG1*, *TOP2A*, *TPX2*, *NUSAP1*, *PBK*, *ASPM*, *RRM2*, and *CDT1* (Fig. 6a). Some of these genes have been verified by qPCR analysis (Supplementary Fig.S2a online). Network cluster 1 for NP cells, on the other hand, exhibited a score of 6.7 and included the following genes: *COL2A1*, *COL9A3*, *MATN3*, *COMP*, *COL11A1*, *ACAN*, *FMOD* (Fig. 6b). These genes were enriched in the matrisome analysis for NP cells. *COL2A1* was found to have the highest number of interactions and was identified as a hub gene. A subset of these genes have been verified by qPCR analysis (Figs. 1b, 2c and Supplementary Fig.S1 online). Taken together, our interaction analysis revealed the structured networks of ECM genes in NP cells.

We next evaluated the transcriptional regulation of gene networks of the AF and NP compartments by querying AF and NP specific genes against the Transcription Factor Checkpoint database to determine the transcription factors that may play role in regulating AF- and NP-specific genes. We identified 31 and 29 transcription factors which were significantly enriched among the genes in AF and NP cells, respectively. The expression level and significance (FDR p-value) of these transcription factors in the AF and NP compartments are shown in (Supplementary Table. S7 and S8 online).

Finally, we examined the transcriptional regulation of genes involved in interaction network of NP and AF compartment. To do this, we identified candidate transcription factors that regulate these genes by performing transcription factor (TF)-gene target regulatory network analysis using iRegulon^34^. For this analysis, we chose to use network Cluster 1 of AF and NP as it appeared the most significant cluster from the above interaction network (Supplementary Table. S5 and S6 online and Fig. 6a,b). The analysis yielded several enriched motifs (normalized enrichment score [NES] > 3) associated with transcription factors in AF and NP. Our analysis identified *FOXM1* and *KDM4E* as novel candidate transcription factors involved in the transcriptional regulation of genes specific to AF and NP, respectively (present in cluster 1). Intriguingly, the ‘regulator-target genes analysis’ demonstrated that *FOXM1* and *KDM4E* potentially target a vast majority of the signature genes of AF and NP, respectively (Fig. 6c,d). *FOXM1* and *KDM4E* mRNA expression was verified by qPCR analysis (Fig. 6e,f).

## Discussion

IDD is a complex multifactorial process influenced by both genetic and environmental factors^2^. The existing clinical strategies are limited in that they only treat the end-stage symptoms rather than address the underlying pathobiology of IDD^1^. Only recently have attempts been made to determine the efficacy of new gene and regenerative stem cell-based therapies for reversal and prevention of IDD progression. However, a better understanding of the transcriptomic signatures of the two IVD compartments is needed to improve the feasibility and effectiveness of these promising strategies. Several studies have made efforts to address this issue, including a study involving bulk RNA sequencing of discectomy-derived NP and AF cells^13^. However, there still remains a gap in our understanding of the gene expression profiles of healthy human AF and NP compartments, as well as the assessment of cell heterogeneity within each compartment. In this study, we have bridged some of these gaps in knowledge by identifying AF- and NP-enriched genes in cells isolated from non-degenerate, healthy human lumbar IVDs. Furtherrmore, we have also mapped the transcriptional landscape of the ECM-related genes in the two compartments of the IVD and identified some of the transcription factors that may regulate the transcriptional profile of AF and NP cells.

One of the key findings of our study is the identification of genes that are compartmentally enriched in either AF or NP cells. The compartment-enriched gene expression of AF markers *CYTL1, ANKRD29*, and *ADGRL4*^13,35^ confirmed the purity of AF cell populations. The higher expression levels of *SFRP1, SFRP4, DKK1, APCDD1L, WNT5A,* in the AF suggests that levels of canonical Wnt signaling may play an important physiological role in maintaining a healthy AF phenotype. Indeed, Wnt/β-catenin signaling was shown to maintain the structure of the AF compartment^36^. The study by Kondo et al. also demonstrated progressive decreases in Wnt/β-catenin signaling in the AF during the transition from E18.5 to 5 weeks of age. Wnt/β-catenin signaling in the NP on the other hand progressively increased from E18.5 to 5 weeks of age^36^. Thus, the compartment-specific expression of canonical Wnt inhibitors such as *SFRP1, SFRP4, DKK1,* and *APCDD1L*^37–40^ may be one of the mechanisms employed to modulate Wnt/β-catenin signaling in the AF. On the other hand, higher expression of *WNT5A* may suggest important roles of non-canonical Wnt signaling^41^ in AF cells. New protective roles of WNT5A have been described in the IVD^42^, but the mechanisms underlying its effects remains to be elucidated. Finally, *ESM1* was also expressed at higher levels in the AF compared to NP cells. This gene is reported to play a role in angiogenesis^43^ and may be responsible for promoting recruitment of blood vessels in the outer AF.

As expected, NP cells expressed hallmark genes that have been previously identified such as *COL2A1*, *KRT8, A2M, CD24, DSC3, COMP, FMOD, ACAN*^26,44–49^ to name a few*,* which were expressed at higher levels compared to AF cells. In addition, we also identified other novel genes such as *ANGPTL7, ISM1,* and *NPR3*. *ANGPTL7* and *ISM1* have been reported to inhibit angiogenesis^50–52^, which may contribute to maintenance of the avascular environment of the healthy NP. *NPR3* may also play an important role in the maintenance and development of the non-degenerative NP as previous studies have shown that mutations in this gene lead to thinning or absence of the NP in mice at postnatal day 21^53,54^*.*

A Recent study has also implicated Wnt/β-catenin signaling to IDD^55^ and showed higher expression of Wnt/β-catenin signaling in IDD as compared to healthy discs^55^. Furthermore, this study shows that conditional activation of β-catenin signaling leads to defects in the AF and NP tissue as well as osteophyte formation in the IVDs of mice^55^. While the IVDs used in our study showed no visible signs of degeneration, our analysis did reveal higher expression of Wnt/β-catenin signaling pathway genes *LRP3, LGR5* and *TCF7L2* in the NP (relative to AF) suggesting that the molecular machinery involved in the initiation of the degenerative process may already be in place much before clinical degeneration occurs later on during the aging process. The role of Wnt/β-catenin signaling in tissue during aging and disease has also been reported previously^56^. Interestingly the NP cells in our study also exhibited higher levels of expression of Wnt/β-catenin signaling inhibitor DKK3^57^, suggesting that healhy NP cells may prevent the onset of degenerative processes by inhibiting Wnt/β-catenin signaling.

Our analysis of core matrisome and matrisome-associated genes allowed us to delve deeper into understanding the ECM landscape of the IVD and differences between the ECM genes expressed by AF and NP cells. We found that 90 of the total 274 core matrisome genes and 115 of the 753 matrisome associated genes were upregulated in the cells of IVD. As such, these genes may play important roles in maintaining the structural integrity of the IVD. Interestingly, we found that NP cells had increased expression of 62 of these core matrisome genes that code for the three major components of the ECM (i.e., ECM glycoproteins, proteoglycans, and collagen). Conversely, AF cells had an increased expression of 28 core matrisome genes, including only 2 proteoglycan genes. The NP is considered the primary site of proteoglycan synthesis in the IVD and contains nearly 50% proteoglycans by dry weight, whereas the AF contains considerable less proteoglycans (approximately 10% by weight)^58^. This data comes in support of our finding of lower expression of proteoglycan genes in AF compared to NP cells. Unlike the core matrisome genes, the total number of highly expressed matrisome-associated genes in NP was only modestly higher than AF cells. However, the number of genes expressing secreted factors were much higher in the NP compared to the AF. The hypocellular nature of the NP compartment may partly explain this finding, as NP cells may rely more heavily on paracrine signaling through secreted factors than AF cells.

Finally, to the best of our knowledge, this is the first study utilizing SCT analysis to evaluate the transcription factors responsible for regulating the NP and AF compartments of healthy human IVDs. We quarried the total number of genes differentially expressed in our study to a transcription factor database to generate a list of transcription factors that are significantly expressed in AF and NP cells. Utilizing interaction network analysis, we show the AF and NP network clusters and the transcription factor-target regulatory network clusters. Our analysis revealed *FOXM1* to be the main transcription factor regulating the AF network clusters, while *KDM4E* was the main transcription factor regulating the NP network clusters. FOXM1 had been reported to play an essential role in the control of cell cycle and mitotic progression^59,60^. *FOXM1* regulates the cell cycle by binding and activating its target genes *CENPF, CCNB1,CCNB2,KPNA2* and *PLK1*^59,60^. Interestingly *FOXM1* has also been implicated in the activation of Survivin (*BIRC5*)^59,60^, a potent anti-apoptotic factor. The identification of FOXM1 and its target genes *CENPF, CCNB1,CCNB2,KPNA2*, *PLK1* and *BIRC5* (Table 1 and Supplementary Table. S1 online) in the healthy AF cells in our SCT analysis suggests an important role of *FOXM1* in maintaining cell numbers and viability in healthy annulus fibrosus cells. An exciting finding of our study is that the NP network cluster consisting mainly of ECM component genes is regulated by *KDM4E*. *KDM4E* is a de-methylase^61,62^ and may play a role in epigenetic regulation of healthy NP cells and their extracellular matrix. While, further studies are required to understand the physiological role of these transcription factors in the context of the IVD, *FOXM1* and *KDM4E* may prove to be a suitable therapeutic target for gene and drug therapy.

Our study not only provides complementary information to the exisiting literature, but also generated new and exciting data on transcriptome signatures of AF and NP cells. This was made possible by the application of an unbiases single cell (sc)RNA-seq approach which has certain advantages over bulk RNA sequencing in the context of the IVD. While the application of bulk RNA sequencing and microarray techniques using whole tissue healthy and degenerated IVDs have provided essential insight into the gross transcriptomic landscape of NP and AF cells in the setting of IDD, certain limitations related to tissue harvesting and bulk analyses should be noted. The high likelihood of contamination with RNA from blood or other cell types may limit the interpretation of the data from potentially heterogeneous subpopulations of cells in the harvested tissue^13^. Moreover, due to the low numbers of cells in these compartments, smaller populations of highly transcriptionally active or repressed cells could influence bulk sequencing results. Similarily, while microarray-based approaches have yielded valuable insight into the gene signatures of IVD tissues^35^, it can be influenced by probe hybridization techniques. It is based on these observations that we acknoweldeged the need for unbiased approaches to assess compartment-specific changes in gene expression between AF and NP starting with a carefully isolated population of cells from each compartment followed by scRNA-seq analysis.

In order to ensure a high degree of homogeneity of AF and NP cells for our analysis, special care was taken to discard the inner transition zone between the AF and NP during the isolation of each compartment (Fig. 1a). The isolated cells were then cultured to obtain a sufficient number of cells for SCT analysis. It is possible that the exclusion of the inner transition zone between the AF and NP may have omitted an important physiological role played by cells in this region. However, we prioritized the need to first have as clean of transcriptomic signatures for AF and NP cells at the single cell level as possible. One could also argue that culturing the cells for expansion may have influenced gene expression during this process. This is certainly a limitation that we recognize and hope to optimize single cell RNA-seq conditions from fresh tissue for future studies. However, one degree of confidence about our data from cultured cells is the confirmation of a variety of compartment specific genes previously identified using bulk RNA-seq experiments from discarded IVD tissues. We have also verified a subset of these marker genes by qPCR analysis (Fig. 1b,c and Supplementary Fig.S1 online). Moreover, t-SNE analysis also confirmed the segregation of AF and NP cells into two discreet and pure populations of cells.

To our knowledge, our study is the first to evaluate the genomic signatures and ECM landscape of NP and AF cells in non-degenerated human IVDs using unbiased scRNA-seq. Herein, we not only provide insight into the genes differentially expressed in the NP and AF compartments, but also examine the transcription factors that may be involved in the regulation of these two compartments. The information obtained from this study may also aid in the development of novel cell- and drug-based regenerative strategies for IDD.

## Methods

### Tissue acquisition and primary cell culture experiments

The study protocol was reviewed and approved by the Emory University Institutional Review Board IRB00099028. All the methods used in this study were carried out in strict adherence with the approved protocol and guidelines. Normal/unaffected disc samples (no recorded history of disc degeneration) from post mortem donors were obtained from the National Disease Research Interchange (NDRI; Philadelphia, PA, USA). Informed consent from legal guardians of post morten donors were obtained regarding the use of their disc tissue for this study, in full compliance with our approved IRB protocol (#00099028). Human IVDs were carefully dissected from L1–L2 to L5–S1 of a 35 year old male donor and 18 and 24 year old female donors. Thompson grading for these samples was evaluated and all samples were of Thompson grade 1 or 2 (Supplementary Fig.S3 and Supplementary Table. S9 online). No fractures or baseline complaints of spinal problems were reported in these donors.

The section of the spine was washed with sterile DPBS (Gibco #14190144) and treated with chlorhexidine for 1 min. The spine was then washed thoroughly with sterile DPBS, followed by careful excision of the IVD with a sterile scalpel. The AF and NP tissues were carefully isolated by removing the inner transition zone between the NP and AF in an attempt to prevent mixing of the cells from the two compartments.

The isolated NP and AF tissue was weighed, homogenized, and treated with proteases (Pronase) for one hour at 37 °C and 5% CO_2_ followed by treatment with collagenases (collagenase P) overnight (12 h) at 37 °C and 5% CO_2_. The cell suspension was then filtered through a 70-μm cell strainer into a 50-mL tube to obtain single NP and AF cells. Cell viability was assessed using the trypan blue dye exclusion test. Primary human AF and NP cells were then seeded at total of 200,000 cells per well of a six-well dish and cultured under normoxic conditions in DMEM/F12 media (Gibco) containing 10% defined FBS and 20 µg/ml ascorbic acid (Sigma) and 1X Pen/Strep (Gibco) for 7–8 days in order to obtain a sufficient number of cells for single-cell transcriptomic analysis. The media was replaced every alternate day. The NP and AF cell identity were confirmed by evaluating the expression of genes that are considered markers of NP and AF cells. Two of the samples (24 year-old female and 35 year-old male) were used for single-cell transcriptomic analysis while the third sample (18 year-old female) was used along with these two samples for verification by qPCR analysis.

### Library preparation

Sequencing libraries were generated using Sure Cell WTA 3′library prep kit (Illumina) following protocol as previously reported^63^. The 3′ mRNA single-cell RNA sequencing libraries were generated utilizing disposable microfluidic cartridges to co-encapsulate single cells and barcodes into sub-nanoliter droplets on a BioRad ddSEQ Single-Cell Isolator. Cell lysis, barcoding, and 3′ RNA Seq libraries generated with an Illumina SureCell WTA 3′ Library Kit were prepared for two NP and two AF samples. The libraries were prepared according to the manufacturer's protocols.The libraries were then purified and sequenced on an Illumina NextSeq 550 with 68 × 75 base pair paired-end reads in mid-output mode.

### Single cell analysis

Sample demultiplexing and gene counts were extracted using the Illumina Sure cell pipeline. The sequencing depth was 100,000 reads per cell and on average 300 cells per experimental group were analyzed using Seurat package in R, version 3.0 (https://satijalab.org/seurat) as described previously^64^. The two samples were sequenced in separate batches. The first batch, from the 24 year old female, contained on average 535 AF and 719 NP cells per sample and 12,230 UMI per cell. The second batch, from the 35 year old male, contained on average 190 AF and 291 NP cells per sample and 10,572 UMI per cell. Downstream analysis was performed with the single-cell consensus clustering tool (SC3)^65^ for cell clustering. Default likelihood ratio tests assuming negative binomial distributions were performed in EdgeR Bioconductor package version 3.11 (https://www.bioconductor.org) to evaluate the significance of differential expression^66^. The pooling strategy creates pseudo cells by pooling groups of 20 cells within each sample and summing their gene count. The gene expression values from the pseudo cells were normalized to counts per million before using EdgeR. Ten permutations of this procedure were performed, and the average differential expression and negative-logP values were computed, and genes significant with a false discovery rate of less than 5% were selected for downstream gene ontology analysis.

### Gene ontology and pathway enrichment analysis

The enrichment analyses for gene ontology (GO) and pathways were performed with ToppFun, which is part of the ToppGene suite^67^. ToppFun evaluates enrichment based on the proportion of genes with a specific functional annotation in the significant list relative to the proportion of those genes in the genome. It similarly evaluates enrichment for genes belonging to known protein interaction networks. FDR adjustments for multiple comparison testing were used to evaluate pathways from BIOCYC, Pathway Interaction Database, REACTOME, GenMAPP, MSigDB C2 BIOCARTA (v6.0), PantherDB, Pathway Ontology, and SMPDB databases. The differentially expressed genes between NP and AF were analyzed for functional enrichment of GO terms and pathways. The GO enrichment was performed for molecular function (MF). MF GO terms were represented by advance bubble chart. The significance of enriched pathways and P-values were calculated based on the cumulative hypergeometric t-test as described previously^68^.

Additionally, we also identified the metabolic genes belonging to Glycolysis, TCA, electron transport chain and pyruvate metabolism, by querrying the highly expressed AF and NP genes against the respective human pathway genes obtained from the RGD database (RGD; https://rgd.mcw.edu)^69,70^. The heatmaps for these metabolic genes was generated using GraphPad Prism version 8.2.0.

### Protein–protein interaction (PPI) network analysis

To determine the biological significance of enriched pathways and associated genes, we performed protein–protein interaction network analysis for the genes expressed at high levels in NP and AF compartments using the STRING (version: 11.0) network analysis^71^. The genes significantly expressed in NP and AF were used as our input gene set, and networks were analyzed for experimentally validated interactions with a combined score of > 0.7 indicating high confidence score for a significant interaction. The nodes lacking a connection in the network were excluded. The interaction network was visualized by Cytoscape (version: 3.7.1), a bioinformatics package for biological network visualization and data integration^72,73^. The CytoNCA plug‐in (version: 2.1.6) was used to analyze the topological properties of nodes in the PPI network, and the parameter was set without weight^74^.

We next performed cluster analysis using the Molecular Complex Detection Algorithm (MCODE) plugin (version: 1.5.1) in Cytoscape^75,76^ to identify the clustering modules in the PPI network. Significant modules were identified according to the clustering score using the following criteria: ‘Degree cutoff = 2’, ‘node score cutoff = 0.2’, ‘Haircut = true’, ‘Fluff = false’, ‘k core = 2’ and ‘max depth = 100’. The clustering modules having high node scores and connectivity degrees were considered as biologically significant clusters. For NP and AF compartment, we chose cluster 1, the most significant cluster and network were visualized with Cytoscape (version: 3.7.1).

### Prediction of regulatory networks of transcription factors (TFs)

Genes identified in the network-cluster 1 for each NP and AF cells were subjected to transcription factor (TF)-target gene regulatory network analysis to identify the candidate transcription factors as described previously^77,78^. We performed iRegulon (version 1.3) analysis using Cytoscape^34^ to examine the transcription factor binding motifs enriched in the genomic regions of our query gene set. The criteria set for motif enrichment analysis were as follows: identity between orthologous genes ≥ 0.0, FDR on motif similarity ≤ 0.001, and TF motifs with normalized enrichment score (NES) > 3. The ranking option for Motif collection was set to 10 K (9,713 PWMs) and a putative regulatory region of 20 kb centered around TSS (7 species) was selected for the analysis. We selected the most significant transcription factor for the extended network analysis for each NP and AF separately.

Additionally, the genes with higher expression levels in NP and AF compartment were queried against the Transcription Factor Checkpoint database (https://www.tfcheckpoint.org/) to identify the number of differentially expressed transcription factors in AF and NP cells. The TFcheckpoint database has a collection of transcription factors that have been annotated as true DNA binding transcription factors (DbTFs) based on experimental and other evidence.

### Comparisons of transcriptome profiles with available microarray datasets

To define the uniqueness of our healthy NP and AF transcriptome and cross-comparison with publicly available datasets, we downloaded the array data generated for non-degenerated NP and AF from several microarray datasets (GSE34095, GSE114169, GSE70362, GSE56081, GSE23130) available in the GEO database. Although these microarray data were performed in degenerated and non-degenerated NP or AF cells, we only retrieved datasets for healthy non-degenerated samples. The GEO datasets included Ref Seq gene ID, gene name, gene symbol, microarray ID, adjusted P-value, and experimental group logFC relative to corresponding control groups. Log fold change (FC) represented the fold change of gene expression and P < 0.05 and logFC > 1 was set for statistically significant DEGs (differentially expressed genes). The global gene dataset comparison of our transcriptomic data with analyzed microarray data was visualized by the Venn diagram and overlap of these two datasets for the global similarities was analyzed by Spearman correlation analysis.

### Analysis of extracellular matrix landscape of NP and AF

We analyzed the extracellular matrix (ECM) landscape of NP and AF in our transcriptome datasets of differentially expressed genes. We queried our transcriptome data against core-matrisome and non-core matrisome datasets (https://matrisomeproject.mit.edu/) to identify the number of differentially expressed matrisome and matrisome-associated genes in each NP and AF compartment. The core matrisome profiling was performed for all three categories-ECM glycoprotein, proteoglycans and collagen. Similarly, the categories used for non-core matrisome profiling were ECM regulator, ECM affiliated protein and secreted factors. The global profiling of core and non-core matrsiome for each NP and AF was visualized by Venn diagram and pie-chart analysis. The differential expression profiling between NP and AF were represented by heatmaps for the expression of core matrisome and matrisome associated genes. The heatmaps for mRNA expression profiling of selected genes was generated using GraphPad Prism version 8.2.0.

### qPCR analysis

Primary human AF and NP cells from an 24-year-old female, 34-year-old male and 18-year-old female donor were plated at a density 200,000 cells of per well of a six-well dish and cultured in DMEM/F12 media containing 10% defined fetal bovine serum, 20 µg/ml ascorbic acid and 1X Pen/Strep for 7–8 days. The media was replaced every alternate day. Total RNA was isolated from AF and NP using TRIzol (Invitrogen) according to the manufacturer’s instructions. cDNA was synthesized using oligo(dT) and random primers with qScript cDNA SuperMix (Quantabio). The Analytik Jena qTower 3 G Real-Time PCR Detection System using PowerUP SYBR green master mix (Applied Biosystems) was used to perform all qPCR reactions. Melt curve analysis was performed to confirm amplicon authenticity. Fold change analysis was conducted using the ∆∆Ct method (Livak & Schmittgen, 2001) and bar diagram was generated using GraphPad Prism version 8.2.0. Primer sequence information is provided in (Supplementary Table 10 online).

## Supplementary information

Supplementary information 1

Supplementary information 2

Supplementary information 3

## Data Availability

The datasets analyzed during the current study are available from the corresponding author on reasonable request.
