# Supplementary material for: Single-cell RNA-seq identifies unique transcriptional landscapes of human nucleus pulposus and annulus fibrosus cells
Source: Sci Rep. 2020 Sep 17;10:15263. doi: 10.1038/s41598-020-72261-7 (PMC7499307; doi:10.1038/s41598-020-72261-7)
Supplement: Supplementary file 1 — Supplementary information 1 [file 41598_2020_72261_MOESM1_ESM.docx]

**Single-cell RNA-seq identifies unique transcriptional landscapes of human** **Nucleus Pulposus and Annulus fibrosus cells**

Lorenzo M. Fernandes^1,2*^, Nazir M. Khan^1,2*^, Camila M. Trochez^3^, Meixue Duan^3^, Martha E. Diaz-Hernandez^1,2^, Steven M. Presciutti^1,2^, Greg Gibson^3^, Hicham Drissi^1,2#^

^1^Department of Orthopaedics, Emory University, Atlanta, GA, USA

^2^Atlanta VA Medical Center, Decatur, GA, USA

^3^Center for Integrative Genomics, Georgia Institute of Technology, Atlanta, GA, USA

*These authors contributed equally

**Supplementary Figure. S1**

**Supplementary Figure. S1. Confirmation of Marker Gene Expression By qPCR analysis:** Relative NP marker gene expression as percentage of expression in AF cells. (n=2 with 3 technical replicates).

**Supplementary Figure. S2**

a

**Supplementary Figure. S2. Confirmation of Gene Expression By qPCR analysis: (a)** Relative gene expression as percentage of expression in NP cells. (b) Relative gene expression as a percentage of AF cells. (n=2 with 3 technical replicates).

b

**Supplementary Figure. S3**

a


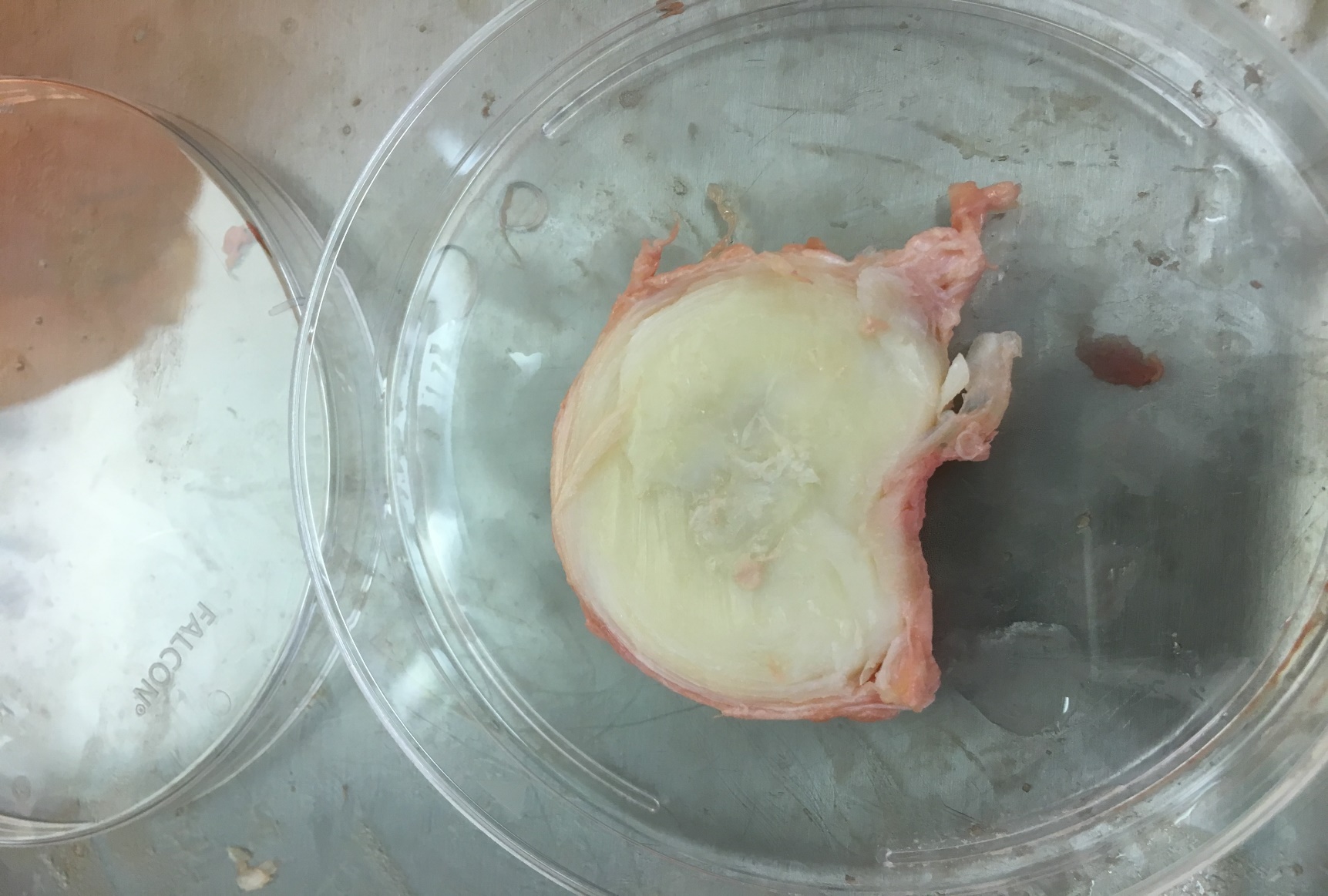


35-Year-old Donor Disc


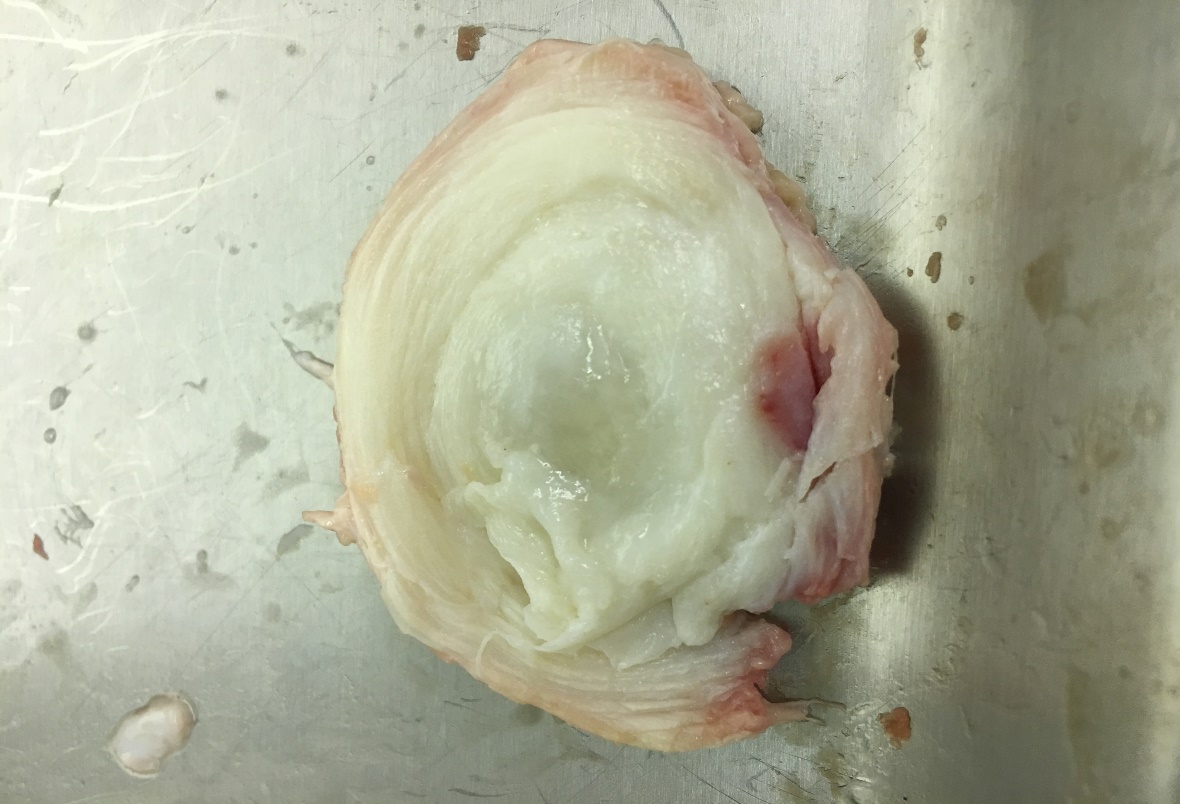


24-Year-old Donor Disc


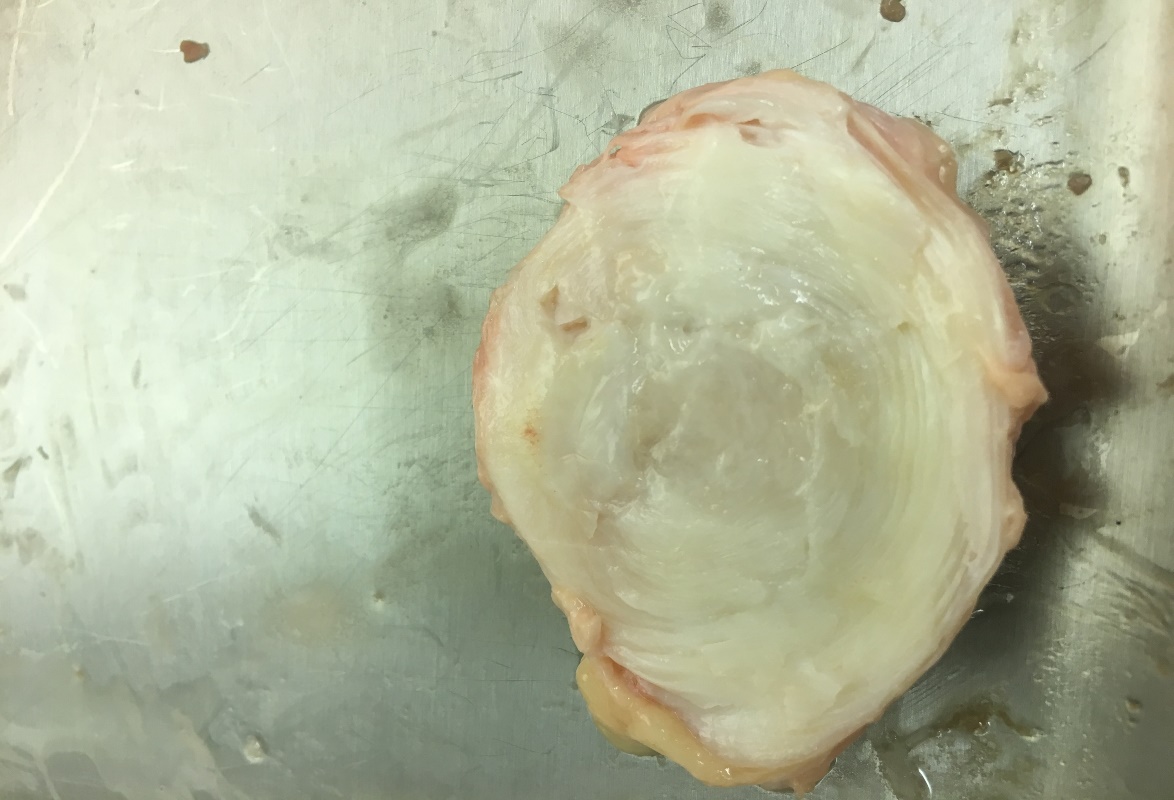


18-Year-old Donor Disc

**Supplementary Figure. S3: Images of Discs used for (sc)-RNA-seq analysis and verification by qPCR. (a)** Images of 24 year old and 35 year old Donor discs used for (sc)-RNA-seq analysis. (b) Image of 18 year old donor disc used for verification of genes by qPCR.

b

**Supplementary Table. S1:** List of genes significantly upregulated in AF as compared to NP (shown in excel sheet).

**Supplementary Table. S2:** List of genes significantly upregulated in NP as compared to AF (shown in excel sheet).

**Supplementary Table. S3: NP Network Topology**

| NP Network Topology | |
| --- | --- |
| Clustering coefficient | 0.206 |
| Connected components | 3 |
| Network diameter | 9 |
| Network radius | 2 |
| Network centralization | 0.177 |
| Shortest path | 3408 (66%) |
| Characteristic path length | 3.3 |
| Avg. number of neighbors | 3.806 |
| Number of nodes | 72 |
| Network density | 0.054 |
| Network heterogeneity | 0.906 |

**Supplementary Table. S3:** Network Topological Characteristic of interaction network enriched in NP

**Supplementary Table. S4: AF Network Topology**

| AF Network Topology | |
| --- | --- |
| Clustering coefficient | 0.393 |
| Connected components | 2 |
| Network diameter | 10 |
| Network radius | 1 |
| Network centralization | 0.211 |
| Shortest path | 8936 (94%) |
| Characteristic path length | 3.849 |
| Avg. number of neighbors | 8.918 |
| Number of nodes | 98 |
| Network density | 0.092 |
| Network heterogeneity | 1.12 |

**Supplementary Table. S4:** Network Topological Characteristic of interaction network enriched in AF

**Supplementary Table. S5: Number of network clusters in AF**

| **Cluster** | **Score (Density*#Nodes)** | **Nodes** | **Edges** | **Node IDs** |  |
| --- | --- | --- | --- | --- | --- |
| 1 | 24.88 | 26 | 311 | DEPDC1, TK1, SHCBP1, CDK1, UBE2C, CCNB2, CCNB1, KIF2C, AURKB, CENPF, CENPE, PLK1, BIRC5, PTTG1, TOP2A, TPX2, FOXM1, NUSAP1, PBK, ASPM, RRM2, CDT1, CENPK, CENPM, UBE2T, KIAA0101 |  |
| 2 | 3.333 | 4 | 5 | SEMA3D, EFNB2, PLXNC1, NTN1 |  |
| 3 | 3 | 5 | 6 | COMP, COL9A3, FMOD, BMP2, ITGA2 |  |
| 4 | 3 | 3 | 3 | LPL, SAA1, APOB |  |

**Supplementary Table. S5:** Table showing number of clusters, cluster scores, nodes (genes) and edges (interactions) in AF cells. Cluster 1 was the most significant cluster with the highest number of nodes and edges.

| **Cluster** | **Score (Density*#Nodes)** | **Nodes** | **Edges** | **Node IDs** |  |
| --- | --- | --- | --- | --- | --- |
| 1 | 6.667 | 7 | 20 | COL2A1, COL9A3, MATN3, COMP, COL11A1, ACAN, FMOD | 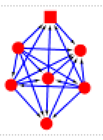 |
| 2 | 3 | 7 | 9 | CP, ACKR3, C3, LPL, IGF1, PPARGC1A, SAA1 |  |

**Supplementary Table. S6: Number of network clusters in NP**

**Supplementary Table. S6:** Table depicting number of clusters, cluster scores, nodes (genes) and edges (interactions) in NP cells. Cluster 1 was the most significant cluster with the largest number of nodes and edges.

**Supplementary Table. S7: Transcription factors expressed at higher levels in the AF cells**

| SN | Gene | Mean FC (Fold change) | Mean FDR  p value |
| --- | --- | --- | --- |
| 1 | *FOXM1* | 6.62 | 8.63E-08 |
| 2 | *TOX2* | 4.97 | 3.80E-04 |
| 3 | *MSX1* | 4.86 | 6.94E-10 |
| 4 | *MAFB* | 4.77 | 1.32E-18 |
| 5 | *HMGA1* | 4.60 | 4.67E-27 |
| 6 | *ETV4* | 4.45 | 8.91E-05 |
| 7 | *MSC* | 3.85 | 1.50E-04 |
| 8 | *SOX4* | 3.57 | 4.85E-19 |
| 9 | *E2F7* | 3.45 | 1.23E-04 |
| 10 | *FOXQ1* | 3.22 | 8.06E-08 |
| 11 | *HMGA2* | 3.20 | 1.38E-09 |
| 12 | *TCF7* | 3.03 | 8.90E-10 |
| 13 | *ETV1* | 2.42 | 1.58E-10 |
| 14 | *MXD3* | 2.37 | 2.22E-03 |
| 15 | *SOX5* | 2.33 | 1.20E-02 |
| 16 | *NR2F1* | 2.17 | 1.57E-05 |
| 17 | *SATB1* | 2.17 | 1.85E-04 |
| 18 | *PRRX1* | 2.08 | 3.31E-17 |
| 19 | *TBX3* | 2.06 | 1.01E-02 |
| 20 | *BHLHE41* | 1.96 | 8.92E-03 |
| 21 | *TEAD2* | 1.93 | 3.59E-03 |
| 22 | *HOXB2* | 1.88 | 1.72E-06 |
| 23 | *HES1* | 1.85 | 8.47E-04 |
| 24 | *SARNP* | 1.81 | 1.06E-11 |
| 25 | *ELK3* | 1.78 | 3.10E-10 |
| 26 | *ETV5* | 1.77 | 1.54E-05 |
| 27 | *LCORL* | 1.73 | 1.29E-02 |
| 28 | *JARID2* | 1.72 | 4.31E-03 |
| 29 | *GPER1* | 1.65 | 9.05E-03 |
| 30 | *ELK1* | 1.63 | 1.22E-03 |
| 31 | *NR2F2* | 1.50 | 7.80E-03 |

**Supplementary Table. S7:** Table showing a list of transcription factors that were found to be significantly expressed at higher levels in the AF cells compared to NP cells by more than 1.5 fold.

**Supplementary Table. S8: Transcription factors expressed at higher levels in the NP Cells**

| SN | Gene | Mean FC | Mean FDR p-value |
| --- | --- | --- | --- |
| 1 | *ZBTB16* | 3.23 | 1.80E-03 |
| 2 | *THRB* | 2.94 | 1.19E-03 |
| 3 | *HIVEP3* | 2.87 | 1.13E-02 |
| 4 | *KLF5* | 2.44 | 4.72E-05 |
| 5 | *ETV7* | 2.43 | 5.05E-03 |
| 6 | *TBX18* | 2.34 | 5.23E-09 |
| 7 | *SNAI1* | 2.28 | 2.31E-04 |
| 8 | *KLF15* | 2.19 | 1.02E-02 |
| 9 | *ZBTB10* | 2.05 | 9.98E-05 |
| 10 | *JDP2* | 2.02 | 9.53E-05 |
| 11 | *MKX* | 1.93 | 5.42E-11 |
| 12 | *THRA* | 1.92 | 2.53E-04 |
| 13 | *SOX15* | 1.88 | 2.76E-02 |
| 14 | *VDR* | 1.77 | 7.82E-05 |
| 15 | *PLAGL1* | 1.68 | 3.08E-06 |
| 16 | *ETS1* | 1.66 | 2.95E-05 |
| 17 | *TCF7L2* | 1.66 | 7.05E-07 |
| 18 | *SIX4* | 1.65 | 4.09E-03 |
| 19 | *CREB3L1* | 1.63 | 3.45E-08 |
| 20 | *SIX2* | 1.61 | 3.90E-03 |
| 21 | *FOXP2* | 1.60 | 4.71E-03 |
| 22 | *PLSCR1* | 1.60 | 2.67E-07 |
| 23 | *KLF4* | 1.56 | 4.74E-06 |
| 24 | *STAT5A* | 1.56 | 1.23E-02 |
| 25 | *KLF2* | 1.55 | 3.40E-03 |
| 26 | *KLF3* | 1.53 | 2.74E-04 |
| 27 | *EBF1* | 1.53 | 6.02E-05 |
| 28 | *STAT1* | 1.52 | 6.40E-08 |
| 29 | *SP110* | 1.50 | 3.93E-03 |

**Supplementary Table. S8:** Table showing a list of transcription factors that were found to be significantly expressed at higher levels in the NP cells compared to AF cells by more than 1.5 fold.

**Supplementary Table. S9: Thompson grade of donor discs**

| N | AGE | Race | SEX | Height (inches) | Weight (KG's) | BMI | Thompson grade of Disc |
| --- | --- | --- | --- | --- | --- | --- | --- |
| 1 | 24 | AA | Female | 70 | 84 | 26.5 | 1 |
| 2 | 35 | C | Male | 76 | 78 | 20.9 | 2 |
| 3 | 18 | C | Female | 63 | 64 | 25.0 | 1 |

**Supplementary Table. S9:** Table showing donor information and Thompson grade of discs.

**Supplementary Table S10. Primers information**

| **GENE NAME** | **NM_ID** | **Forward Primer** | **Reverse Primer** |
| --- | --- | --- | --- |
| *LRRC17* | NM_001031692.3 | GCATCCATTCCCCAAGTTCAG | GCCCCAATTACTTCGTGTTAC |
| *AK5* | NM_012093.4 | ACATTATGGAACGTGGAGACC | AACTCTTCCCCTTGCTTCAC |
| *SFRP1* | NM_003012.5 | AAGTGTGACAAGTTCCCCG | TGGCCTCAGATTTCAACTCG |
| *CYTL1* | NM_018659 | TGAAGGACAAAGCACGGAAG | GTAGTCACTGGGATTGGGTATTC |
| *PCLAF*  *(KIAA0101)* | NM_001029989 | AAGCAGACAGTGTTCCAGG | GTGTGATCAGGTTGCAAAGG |
| *COL11A1* | NM_001854 | TTGGTGTTGAGGTTGGGAG | TTCTCCACGCTGATTGCTAC |
| *DSC3* | NM_001941 | TCTATTTTGCACTCGGCCTG | TGTCATTTTCATCCTCTACCCTG |
| *COL2A1* | NM_001844 | AAGACGTGAAAGACTGCCTC | TTCTCCTTTCTGTCCCTTTGG |
| *COL9A3* | NM_001853 | TTCAGTGCCCAAGTATCTGC | TCGCCTTTGTAGCCAGTG |
| *FAM46B* | NM_052943 | AGCCGATTCCCATTCACG | GCACATGTAGTCCCTGCTC |
| *FOXM1* | NM_202003 | AGAATTGTCACCTGGAGCAG | TTCCTCTCAGTGCTGTTGATG |
| *KDM4E* | NM_001161630 | TCATCTCGCCTACAGTTCTCA | GTTGAAGCCGTGATTGAAGC |
| *ACAN* | NM_001135 | TGTGGGACTGAAGTTCTTGG | AGCGAGTTGTCATGGTCTG |
| *FMOD* | NM_002023 | CTCTCACACGTTCTCCAACC | GTCATCTTCATACTGGGCCTG |
| *COMP* | NM_000095 | GTACCCAACTCAGACCAGAAG | TCACAAGCATCTCCCACAAAG |
| *MSX1* | NM_002448.3 | TCAAGCTGCCAGAAGATGC | TGTGTTTGCGGAGGGTG |
| *EDNRA* | NM_001957 | TGCCCTCAGTGAACATCTTAAG | CATCGGTTCTTGTCCATCTCG |
| *PLK1* | NM_005030 | ACAGTTTCGAGGTGGATGTG | GGTTGATGTGCTTGGGAATAC |
| *CDK1* | NM_001786 | ACAAAGGAACAATTAAACTGGCTG | CTGGAGTTGAGTAACGAGCTG |
| *BIRC5* | NM_001012270 | CTTCATCCACTGCCCCAC | ACTTTCTCCGCAGTTTCCTC |
| *C2ORF82*  *(SNORC)* | NM_206895 | TTAACCAGCGCAGTCCTC | GGCTCCTGTGGAACATCG |
| *SAA1* | NM_000331.5 | TTTTCTGCTCCTTGGTCCTG | TGGAAGTATTTGTCTGAGCCG |
| *EFHD1* | NM_025202 | ACTTCGATGGCAAGCTCAG | CACCTTTGACACCCTCCAG |
| *IL15* | NM_000585.5 | ACCGTGGCTTTGAGTAATGAG | AAGCCCTGCACTGAAACA |
| *PCSK2* | NM_002594 | CTGTGGAGTTGGAGTAGCATAC | TGGCGCTGTAGATGTCAATC |
| *ISM1* | NM_080826 | TGAAACCAAAGATCAGCCAGA | TCCTCGATTCTGTTGCAGTG |

**Supplementary Table S10:** Primers sequences (Forward and Reverse) used in PCR
